# Prevalence of Comorbidity between Dry Eye and Allergic Conjunctivitis: A Systematic Review and Meta-Analysis

**DOI:** 10.3390/jcm11133643

**Published:** 2022-06-23

**Authors:** Yasutsugu Akasaki, Takenori Inomata, Jaemyoung Sung, Masahiro Nakamura, Koji Kitazawa, Kendrick Co Shih, Takeya Adachi, Yuichi Okumura, Kenta Fujio, Ken Nagino, Akie Midorikawa-Inomata, Mizu Kuwahara, Kunihiko Hirosawa, Tianxiang Huang, Yuki Morooka, Hurramhon Shokirova, Atsuko Eguchi, Akira Murakami

**Affiliations:** 1 Department of Ophthalmology, Juntendo University Graduate School of Medicine, Tokyo 1130033, Japan; y-akasaki@juntendo.ac.jp (Y.A.); jsung1@usf.edu (J.S.); masahiro-nakamura@umin.ac.jp (M.N.); y-okumura@juntendo.ac.jp (Y.O.); k.fujio.zz@juntendo.ac.jp (K.F.); mz-ohno@juntendo.ac.jp (M.K.); k-hirosawa@juntendo.ac.jp (K.H.); h.tianxiang.zb@juntendo.ac.jp (T.H.); y.morooka.df@juntendo.ac.jp (Y.M.); h-shokirova@juntendo.ac.jp (H.S.); amurak@juntendo.ac.jp (A.M.); 2 Department of Digital Medicine, Juntendo University Graduate School of Medicine, Tokyo 1130033, Japan; k-nagino@juntendo.ac.jp; 3 Department of Hospital Administration, Juntendo University Graduate School of Medicine, Tokyo 1130033, Japan; ak-inomata@juntendo.ac.jp (A.M.-I.); a-eguchi@juntendo.ac.jp (A.E.); 4 AI Incubation Farm, Juntendo University Graduate School of Medicine, Tokyo 1130033, Japan; 5 Precision Health, Department of Bioengineering, Graduate School of Engineering, The University of Tokyo, Tokyo 1138656, Japan; 6 Department of Ophthalmology, Kyoto Prefectural University of Medicine, Kyoto 6028566, Japan; kkitazaw@koto.kpu-m.ac.jp; 7 Buck Institute for Research on Aging, Novato, CA 94945, USA; 8 Department of Ophthalmology, Li Ka Shing Faculty of Medicine, The University of Hong Kong, Hong Kong, China; kcshih@hku.hk; 9 Department of Dermatology, Keio University School of Medicine, Tokyo 1608582, Japan; jpn4156@me.com

**Keywords:** allergic conjunctivitis, dry eye, systematic review, meta-analysis, seasonal allergic conjunctivitis, perennial allergic conjunctivitis, comorbidity rate, complementary treatment

## Abstract

This systematic review aimed to determine the comorbid dry eye (DE) and allergic conjunctivitis (AC) prevalence. We searched PubMed and EMBASE for articles published until 22 March 2022, combining the terms “(dry eye OR keratoconjunctivitis sicca) AND allergic conjunctivitis.” Study-specific estimates (DE and AC incidence rates among patients with AC and DE, respectively) were combined using the one-group meta-analysis in a random-effects model. The initial search yielded 700 studies. Five articles reporting AC incidence among individuals with DE and six articles reporting DE incidence among individuals with AC were included in the qualitative synthesis. In these nine articles, the total sample size was 7254 patients. The DE incidence among individuals with AC was 0.9–97.5%; the AC incidence among individuals with DE was 6.2–38.0%. One-group meta-analysis using a random-effects model showed that 47.2% (95% confidence interval: 0.165–0.779; 320/1932 cases) of patients with AC had comorbid DE and 17.8% (95% confidence interval: 0.120–0.236; 793/4855 cases) of patients with DE had comorbid AC, as defined by each article. Complimentary screening and treatment for patients with DE and AC may improve long-term outcomes and prevent chronic ocular damage in highly susceptible populations.

## 1. Introduction

Dry eye (DE) and allergic conjunctivitis (AC) are the two most common ocular surface diseases [1,2,3] that negatively affect one’s quality of life and work productivity [4,5]. DE and AC, particularly seasonal AC (SAC) and perennial AC (PAC), present with a wide range of symptoms that significantly overlap, such as itching, redness, and dryness [6]. Moreover, both diseases are highly multifactorial in terms of onset and aggravation and share a considerable number of risk factors [1,5,7]. Currently, the known risk factors for the two diseases include female sex, history of contact lens use, presence of allergic diseases, and hay fever [2,5,8,9]. As DE and AC share common ocular symptoms and risk factors, proper diagnosis and treatment of these diseases and their comorbidities are important, with tailored regimens for each of the three states.

The fundamental pathophysiology of DE and AC is rooted in the immunological alterations that result in inflammation of the ocular surface, and their shared pathogenesis paves the way to negative synergies that aggravate the other diseases [10,11,12]. Previous reports have suggested that the reduced tear volume caused by DE hinders the removal of allergenic antigens on the ocular surface in patients with hay fever, which exacerbates AC associated with hay fever [13,14]. Similarly, AC has been shown to disrupt the tear film stability, contributing to worse outcomes in patients with DE [13,14]. These negative interactions between the two diseases necessitate bidirectional diagnosis and management to prevent chronic damage to the ocular surface. However, to our knowledge, no study to date has described the differentiating characteristics and prevalence of these two diseases as comorbidities.

Therefore, we conducted this systematic review to identify the prevalence of comorbid DE and AC, including SAC and PAC.

## 2. Materials and Methods

### 2.1. Outcomes

The primary aim of this study was to systematically evaluate and characterize the current reports of DE and AC, including SAC and PAC. In particular, the primary analysis focused on the comorbidity rates of DE and AC.

### 2.2. Search Strategy

This study was conducted in accordance with the Preferred Reporting Items for Systematic Reviews and Meta-Analyses reporting guidelines [15]. An extensive search strategy was designed to retrieve all articles published until 22 March 2022, in PubMed and EMBASE, which are key electronic bibliographic databases, by combining the terms “(dry eye OR keratoconjunctivitis sicca) AND allergic conjunctivitis.” The inclusion and exclusion criteria for the study are listed in Table 1. Search results were compiled using EndNote X9.3.3 software (Clarivate Analytics, Philadelphia, PA, USA). In accordance with the quality standards for reporting systematic reviews and meta-analyses of observational studies [16], two independent researchers (Y.A. and T.I.) screened the retrieved articles. The same investigators independently assessed the full text of records deemed eligible by consensus.

### 2.3. Data Extraction

Two independent reviewers (Y.A. and T.I.) extracted data from each eligible study using a standardized data extraction sheet and subsequently cross-checked the results. Disagreements regarding the extracted data were resolved through discussion with a third reviewer (K.K.) [17]. Extracted data included the first author’s name; date of publication; type of study (retrospective or prospective); country of study; cohort size; characteristics of patients with DE and AC, including their age and sex; and the incidence rates of DE and AC among individuals with AC and DE, respectively.

### 2.4. Statistical Analysis

Study-specific estimates (incidence rate of DE among individuals with AC and incidence rate of AC among individuals with DE) were combined using the one-group meta-analysis in a random-effects model using OpenMetaAnalyst version 12.11.14 (Available from http://www.cebm.brown.edu/openmeta/, accessed on 22 June 2022) [18].

## 3. Results

**Figure 1:**
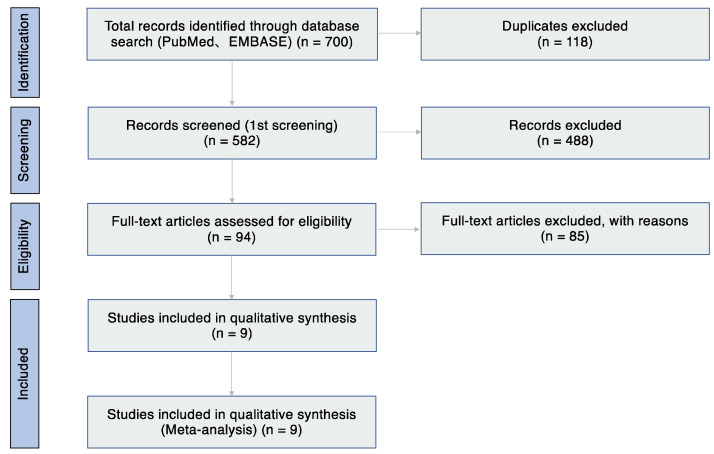
Flow diagram of study selection.

The database search identified 700 articles. After removing 118 duplicates, 582 articles were reviewed based on the title and abstract. Of these articles, 488 studies were excluded because of the article type (clinical guidelines, consensus documents, reviews, and conference proceedings) and because they focused on other subtypes of AC (atopic keratoconjunctivitis, vernal keratoconjunctivitis, giant papillary conjunctivitis, and atopic blepharitis), had an animal-based design, focused on other unrelated topics and drug allergy, and were not written in English (Table 1). Thus, 94 articles were assessed for eligibility (Figure 1), and eventually, studies on nine studies met the inclusion criteria and were included in the systematic review (Table 2).

### 3.1. Study Characteristics and Demographic Features

The articles included in this systematic review were published between 2012 and 2021 (Table 2). Four studies were from Japan [20,21,22,26], while one each was from Thailand [19], China [23], Turkey [24], Nigeria [25], and Ghana [27]. Seven were prospective studies [19,20,21,22,23,24,26], and two were retrospective studies [25,27]. The total sample size was 7254 patients. Three studies primarily included children [19,23,24]. The diagnostic examination for DED used in those included articles were shown in Table 3. Seven articles reported the mean age [19,21,22,23,24,26,27], and the mean age range of the included adults was 30.6 [27] to 69.1 [21] years, whereas the mean age range of children was 4.75 [23] to 11.79 [24] years. All articles mentioned the sex of the patients; there were 1892 men and 5362 women [19,20,21,22,23,24,25,26,27].

### 3.2. Incidence DE among Individuals with AC

Six articles reported the incidence of DE among individuals with AC [19,21,23,24,25,27], which ranged from 0.9% [25] to 97.5% [23]. Among these articles, 16.6% (320/1932 cases) of individuals with AC had DE. Three articles reported the incidence of DE among individuals with AC (110/158 patients (69.9%) [21], 9/972 eyes (0.9%) [25], and 126/696 patients (18.1%) [27]). Three articles reported the incidence of DE among children with AC (30/41 patients (73.2%) [19], 78/80 eyes (97.5%) [23], and 6/25 patients (24.0%) [24]). Two studies reported DE examinations findings and compared the findings from the AC group with those from the control group [23,24]. Chen L et al. [23] reported that the tear film break-up time (TFBUT) was lower in the AC children group (6.54 ± 1.48 s) than in the control group (10.04 ± 1.79 s; *p* < 0.001), and there was a negative correlation between the papillary reaction and the Schirmer test, TFBUT, and tear meniscus height reflex (TMH-R) values (r = −0.454, −0.412, and −0.419; *p* = 0.001, 0.003, and 0.002, respectively). However, Akil et al. [24] reported that the values of the Schirmer test, TFBUT, and TMH-R were lower in the AC than in the control group (*p* < 0.001 for all comparisons), and there was a negative correlation between the duration of AC and TFBUT (r = −0.45; *p* < 0.005).

**Figure 2:**
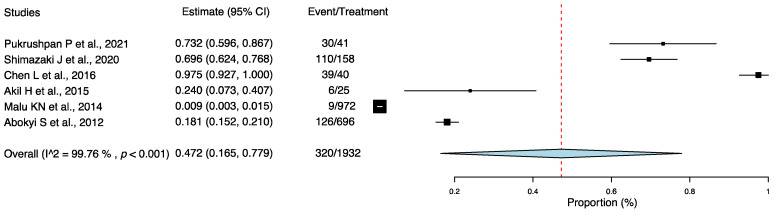
Incidence rate of dry eye among individuals with allergic conjunctivitis. Abbreviation: 95% CI: 95% confidence interval [19,21,23,24,25,27].

Using the one-group meta-analysis in a random-effects model, six studies that included 1932 individuals were further analyzed for the incidence rate of DE among individuals with AC (Figure 2); the results revealed that 47.2% (95% confidence interval: 0.165–0.779; 320/1932 cases) of patients with AC had DE.

### 3.3. Incidence of AC among Individuals with DE

Five studies reported the incidence of AC among individuals with DE [19,20,21,22,26], which ranged from 6.2% [22] to 38.0% [19]. Among these articles, 16.3% (793/4855 cases) of individuals with DE had AC. Four articles reported the incidence of AC among individuals with DE (97/580 (16.7%) [20], 110/551 (20.0%) [21], 28/449 (6.2%) [22], and 528/3196 patients (16.5%) [26]). These four studies were multicenter studies in Japan, two of which evaluated the safety and efficacy of 3% diquafosol [20,26]. One article reported the incidence of AC among children with DE (30/79 patients (38.0%) [19]).

**Figure 3:**
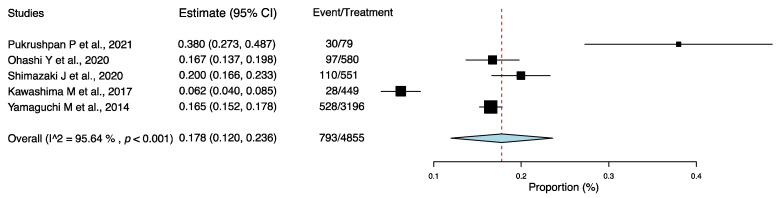
Incidence rate of allergic conjunctivitis among individuals with dry eye. Abbreviation: 95% CI: 95% confidence interval [19,20,21,22,26].

Using the one-group meta-analysis in a random-effects model, five studies that included 4855 subjects were further analyzed for the incidence rate of AC among individuals with DE (Figure 3); the results revealed that 17.8% (95% confidence interval: 0.120–0.236; 793/4855 cases) of patients with DE had AC.

## 4. Discussion

DE and AC are inflammatory diseases of the ocular surface that exhibit a synergistic effect in terms of pathology. In this study, we performed a systematic review and meta-analysis of the prevalence of comorbid DE and AC, including SAC and PAC. Our results indicated that comorbid DE and AC existed in almost half of the patients with AC and DE, respectively. Cautious diagnosis and bidirectional management of the two diseases, with due consideration of their existence as comorbidities, can improve the long-term outcomes.

This study found that the incidence rates of DE and AC among individuals with AC and DE were 47.2% and 17.8%, respectively. Previous studies on the epidemiology of DE have reported a global prevalence of 5–50% [1,28], and the relatively higher comorbidity rate in the AC cohort suggests that patients with AC may be predisposed to DE. Currently, the proposed pathological mechanisms include AC-induced lipid layer thickening of the tear film [14], instability of the tear film caused by the tear protein changes in AC [29], increased inflammatory cytokines secondary to a chronic disease [12,30], and prolonged use of antihistamines for symptomatic management [27,31], all of which may contribute to tear film disruption and the pathogenesis of DE [23,32]. In addition, studies have reported that AC may induce squamous metaplasia of the conjunctival epithelium and decrease conjunctival goblet cell density [33]. In an animal study, repeated exposure to allergens in a mouse model of AC showed a similar decrease in the number of conjunctival goblet cells, with a decrease in Muc5AC and Muc4 mRNA expression [34]. Damage to the goblet cells and altered mucin production can exacerbate the existing DE pathology [35,36], implying a direct link between AC and short-TFBUT-type DE [14,36]. The decreased tear production volume also hinders the removal of antigens from the ocular surface, creating a vicious cycle [13]. However, there are conflicting reports on the effects of AC on the clinical findings associated with DE. A study on patients with DE and mild conjunctivitis, including AC, revealed no significant effect of conjunctivitis on TFBUT [13]. Conversely, a study noted a significant decrease in TFBUT in patients with SAC [14]. In a study investigating the effects of AC and non-AC in a pediatric cohort, the AC group exhibited a decreased tear production volume, TFBUT, and central reflex tear meniscal height [24]. This finding indicates that various factors, such as the patient’s age and AC severity, may affect the comorbidity rates of AC and DE.

Contrarily, the incidence of DE among individuals with AC was presumed to be higher than the prevalence among the general population as the decreased tear production volume because DE hinders the removal of antigens from the ocular surface [13]. However, our study found that the incidence of DE among individuals with AC was 17.8%, and there was no significant difference compared with a previous report, which reported a prevalence of AC ranging from 15% to 20% [37,38]. This observation may be attributed to variations in the sample size, sampling bias, and differences in the diagnostic criteria for DE and AC.

Our results indicate that insufficient management of either disease in a comorbid patient could accelerate ocular damage and worsen the prognosis. This calls for a careful diagnosis and management with due consideration of their existence as comorbidities in patients exhibiting these conditions. Unfortunately, AC and DE have a wide range of overlapping manifestations, from eye dryness to itching, and clinicians must conduct detailed and comprehensive examinations to aid in their differentiation [6]. Determining any shared risk factors, including hay fever and contact lens use, is crucial during the initial evaluation [2,5,8,10,11]. In all patients with DE, the history of allergic diseases should be additionally investigated, and the findings of palpebral erythema and edema should be noted. Similarly, Schirmer test results, TFBUT, and tear film break patterns [39] should be examined in patients with AC to rule out the possibility of comorbid DE. To prevent DE onset in patients with AC, extensive and personalized strategies to avoid allergens should be implemented [40,41], starting with an allergen test to determine the causal allergenic factors.

An effort to promote societal education recommending appropriate clinical examinations for DE and AC may have a strong influence on the outcome of their management [5,7]. A study on DE and its association with video display terminal use revealed a higher DE rate among the working-age population, with existing concerns for a high prevalence of AC among the same population [40,41,42]. Recent findings also raised concerns regarding a high percentage of missed diagnoses of chronic DE in the working-age population [43]. By reaching such critical and wide populations in a timely manner, medicine driven by smartphone-based mHealth applications appears to hold potential in identifying symptoms and individual risk factors and in recommending appropriate management and hospital visits [40,41,43,44,45,46,47,48,49].

Missed diagnosis of DE in patients with AC may exacerbate DE and affect the long-term prognosis, as symptomatic management of AC through antihistamines can induce decreased tear production, with supporting evidence from a previous animal study [50]. Management of AC should be appropriately adjusted following a comprehensive examination to determine the presence of DE [51]. Such examinations include the Schirmer test and fluorescein dye examination, which focus on tear production and quality, and are relatively specific for DE diagnosis. Then, the results can be used to determine the need for tear replenishment or invasive techniques, such as punctal plug insertion and cautery. There have been a few reports describing the risk factors for comorbid DE and AC [27] and optimal treatment for comorbid DE and AC. Hence, further research is needed to establish appropriate management for patients with AC and DE.

This study had a few limitations. First, there was a relatively high variation in the sample size per study among the included publications. Second, compared with the sample size in the prevalence studies independently conducted for either DE or AC, the total sample size in the present study might be considered small [1,37,52]. Third, sampling bias concerning limited geographic coverage may be present, as a significant portion of the included studies were conducted in Japan and other Asian countries [19,20,21,22,23,26]. Fourth, there were discrepancies in the diagnostic criteria for DE and AC, as well as varied individual characteristics, such as age, in each included study. In addition, symptoms of DE and AC can be difficult to distinguish because of the symptoms overlap. Future studies concerning the epidemiology of comorbid DE and AC should establish and apply standardized criteria for these two diseases in a larger sample size.

In conclusion, our study results suggest that almost half of the patients with AC may exhibit comorbid DE, and almost 20% of patients with DE may exhibit comorbid AC. This warrants careful examinations for the counterpart disease in patients presenting with either disease, followed by appropriate modification of treatment regimens to minimize the exacerbation of AC and DE.

## Figures and Tables

**Table 1:** Study inclusion and exclusion criteria.

**Inclusion Criteria**	
1	Population: patients with dry eye and seasonal allergic conjunctivitis or perennial allergic conjunctivitis
2	Study design: retrospective (cross-sectional studies, case-control studies, case series, and case reports) and prospective studies
3	Outcome: evaluation of incidence of DE among individuals with AC or incidence of AC among individuals with DE
**Exclusion Criteria**	
1	Clinical guidelines, consensus documents, reviews, and conference proceedings
2	Studies regarding other subtypes of allergic conjunctivitis (i.e., atopic keratoconjunctivitis, vernal keratoconjunctivitis, giant papillary conjunctivitis, and atopic blepharitis)
3	Animal-based studies
4	Unrelated topics
5	Articles not published in English
6	Drug allergy

Abbreviations: DE, dry eye; AC, allergic conjunctivitis.

**Table 2:** Results of systematic reviews on the association between dry eye and allergic conjunctivitis.

Author (Year)	Country	Study Type	Study Population			DE			AC			AC/DE	DE/AC	Findings
			Number	Age (SD)	Sex: M/F	Number	Age (SD)	Sex: M/F	Number	Age (SD)	Sex: M/F			
Pukrushpan P et al. [19] (2021)	Thailand	PS	* Study: 100 (patients) Control: 100 (patients)	* Study: 7.9 (2.0) Control: 9.5 (2.3)	* Study: 77/23 Control: 44/56	* Study: 73 (patients) Control: 6 (patients)	NA	NA	* Study: 41 (patients) Control: 0 (patients)	NA	NA	38.0% (30/79)	73.2% (30/41)	DE, AC, and tic disorder were the commonly associated disorders with excessive blinking in children.
Ohashi Y et al. [20] (2020)	Japan	PS	580 (patients)	NA (NA)	99/481	580 (patients)	NA	99/481	97 (patients)	NA	NA	16.7% (97/580)	NA	Diquafosol 3.0% ophthalmic solution was effective for the treatment of DE.
Shimazaki J et al. [21] (2020)	Japan	PS	990 (patients)	69.1 (13.4)	384/606	551 (patients)	NA	NA	DE: 110 (patients) Non-DE: 48 (patients)	NA	NA	20.0% (110/551)	69.9% (110/158)	More than half of the outpatients had DE, and patients with AC had a high prevalence of DE.
Kawashima M et al. [22] (2017)	Japan	PS	449 (patients)	62.6 (15.7)	63/386	449 (patients)	62.6 (15.7)	63/386	28 (patients)	NA	NA	6.2% (28/449)	NA	Aqueous-deficient and short-TFBUT-type dry eye were the two most common DE subtypes.
Chen L et al. [23] (2016)	China	PS	AC: 40 (patients) control: 40 (patients)	AC: 4.75 (0.83) Control: 4.76 (0.86)	AC: 23/17 Control: 21/19	AC: 78 (eyes) Control: 22 (eyes)	NA	NA	40 (patients)	4.75 (0.83)	23/17	NA	97.5% (78/80)	The incidence rate of DE was higher among young children with SAC and PAC than among controls.
Akil H et al. [24] (2015)	Turkey	PS	AC: 25 (patients) Control: 24 (patients)	AC: 11.18 (2.5) Control: 10.94 (4.3)	23/26	AC: 6 (patients) Control: NA	NA	NA	25 (patients)	11.18 (2.5)	NA	NA	24.0% (6/25)	Children with AC had lower Schirmer test, TFBUT, and TMH-R values.
Malu KN et al. [25] (2014)	Nigeria	RS	972 (patients)	NA	NA	9 (patients)	NA	NA	972 (patients)	22 (NA)	474/498	NA	0.9% (9/972)	AC was the most common eye condition observed in an outpatient clinic in Jos, the capital of Plateau state in North Central Nigeria.
Yamaguchi M et al. [26] (2014)	Japan	PS	3196 (patients)	62.4 (NA)	456/2740	3196 (patients)	62.4 (NA)	156/2740	528 (patients)	NA	NA	16.5% (528/3196)	NA	Diquafosol was effective for various patients with DE without concerns regarding safety.
Abokyi S et al. [27] (2012)	Ghana	RS	738 (patients)	30.6 (16.9)	228/510	129 (patients)	NA (NA)	NA	696 (patients)	NA	NA	NA	18.1% (126/696)	Patients with allergic conjunctivitis disease (SAC + PAC + atopic keratoconjunctivitis + vernal keratoconjunctivitis) treated with systemic antihistamines had a high risk of DE.

* Study group: Children with a chief complaint of excessive blinking. Abbreviations: DE, dry eye; AC, allergic conjunctivitis; SAC, seasonal allergic conjunctivitis; PAC, perennial allergic conjunctivitis; TFBUT, tear film break-up time; TMH-R, tear meniscus height reflex; IQR, interquartile range; NA, not applicable; PS, prospective study; RS, retrospective study; SD, standard deviation.

**Table 3:** Diagnostic examinations for DE.

Author (Year)	Diagnostic Examination for DE
Pukrushpan P et al. [19] (2021)	TFBUT
Ohashi Y et al. [20] (2020)	DEQS, TFBUT
Shimazaki J et al. [21] (2020)	DEQ-5, TFBUT, DE history in the past 2 years
Kawashima M et al. [22] (2017)	TFBUT, Schirmer I test, kerato-conjunctival staining score, and dry eye symptom questionnaire score
Chen L et al. [23] (2016)	TFBUT
Akil H et al. [24] (2015)	Ocular Surface Disease Index, TFBUT, TMH-R, SchirmerⅠtest
Malu KN et al. [25] (2014)	NA
Yamaguchi M et al. [26] (2014)	NA
Abokyi S et al. [27] (2012)	Patients’ symptoms, TFBUT

Abbreviations: TFBUT, tear film break-up time; DEQS, dry eye-related quality of life score; DEQ5, 5-item dry eye questionnaire; DE, dry eye; TMH-R, tear meniscus height reflex; NA, not applicable.

## Data Availability

All data generated or analyzed during this study are included in this published article.

## Abbreviations

DE, dry eye; AC, allergic conjunctivitis; SAC, seasonal allergic conjunctivitis; PAC, perennial allergic conjunctivitis; TFBUT, tear film break-up time; TMH-R, tear meniscus height reflex.
